# Retraction: Transcriptome profiling reveals new insights into the roles of neuronal nitric oxide synthase on macrophage polarization towards classically activated phenotype

**DOI:** 10.1371/journal.pone.0260912

**Published:** 2021-12-17

**Authors:** Pingan Chang, Hao Gao, Quan Sun, Xiaohong He, Feifei Huang

After this article [1] was published, the corresponding author requested its retraction because the transcriptome data belongs to a third party and was analysed and published without permission of the data owner.

In light of this, the *PLOS ONE* Editors and the authors retract this article.

PC, HG, QS, XH and FH agreed with retraction. PC, HG, QS, XH and FH apologized for the issues with the published article.

At the time of retraction, all the article’s supporting information files were removed in light of the data ownership issue. The article’s Data Availability statement and in-text citations of the relevant files were likewise updated.

## References

[1] Chang P, Gao H, Sun Q, He X, Huang F (2021) Transcriptome profiling reveals new insights into the roles of neuronal nitric oxide synthase on macrophage polarization towards classically activated phenotype. PLoS ONE 16(9): e0257908. 10.1371/journal.pone.0257908 34587205 PMC8480887

